# Pathways to cellular supremacy in biocomputing

**DOI:** 10.1038/s41467-019-13232-z

**Published:** 2019-11-20

**Authors:** Lewis Grozinger, Martyn Amos, Thomas E. Gorochowski, Pablo Carbonell, Diego A. Oyarzún, Ruud Stoof, Harold Fellermann, Paolo Zuliani, Huseyin Tas, Angel Goñi-Moreno

**Affiliations:** 1 School of Computing, Newcastle University, Newcastle Upon Tyne, NE4 5TG UK; 2 Department of Computer and Information Sciences, Northumbria University, Newcastle upon Tyne, NE1 8ST UK; 3 BrisSynBio, University of Bristol, Tyndall Avenue, Bristol, BS8 1TQ UK; 4 School of Biological Sciences, University of Bristol, Tyndall Avenue, Bristol, BS8 1TQ UK; 5 Manchester Synthetic Biology Research Centre for Fine and Speciality Chemicals (SYNBIOCHEM), Manchester Institute of Biotechnology and School of Chemistry, University of Manchester, Manchester, M1 7DN UK; 6 School of Informatics, University of Edinburgh, Edinburgh, EH8 9AB UK; 7 School of Biological Sciences, University of Edinburgh, Edinburgh, EH9 3BF UK; 8 Systems and Synthetic Biology Program, Centro Nacional de Biotecnologí­a (CNB-CSIC), Campus de Cantoblanco, 28049 Madrid, Spain

**Keywords:** Synthetic biology, DNA computing and cryptography

## Abstract

Synthetic biology uses living cells as the substrate for performing human-defined computations. Many current implementations of cellular computing are based on the “genetic circuit” metaphor, an approximation of the operation of silicon-based computers. Although this conceptual mapping has been relatively successful, we argue that it fundamentally limits the types of computation that may be engineered inside the cell, and fails to exploit the rich and diverse functionality available in natural living systems. We propose the notion of “cellular supremacy” to focus attention on domains in which biocomputing might offer superior performance over traditional computers. We consider potential pathways toward cellular supremacy, and suggest application areas in which it may be found.

## Abstract (web summary)

Synthetic biology uses cells as its computing substrate, often based on the genetic circuit concept. In this Perspective, the authors argue that existing synthetic biology approaches based on classical models of computation limit the potential of biocomputing, and propose that living organisms have under-exploited capabilities.

## Introduction

Synthetic biology^1,2^ is a rapidly growing field of research which applies engineering concepts and principles to the rational engineering of living systems, such as bacteria and yeast. The promise of synthetic biology lies in its potential to provide new substrates for computation^3^, production^4^, pollution control^5^ and medical diagnosis^6^ (among many areas), and to harness the “wetware”^7^ inside the living cell for human-defined purposes. Synthetic biology is set to become a significant component of the multi-billion dollar bio-economy^8^, but, in addition to tangible benefits such as cheaper drug production or more precise bio-sensing, many researchers in the field believe that the very process of re-engineering life will both require and inform a reexamination of our fundamental understanding of cellular processes^9^. This position underpins the current paper.

Much existing work in synthetic biology is concerned with the construction of “circuits” of biological components (such as genes and proteins) that receive some input (either endogenous, or exogenous), perform some transformation of that input, and produce a result determined by the “rules” encoded in the circuit. This input-transform-output pipeline is entirely consistent with the notion of a computation, as traditionally defined, and it is wholly unsurprising that synthetic biology has, so far, largely adhered to the genetic “logic circuit” model (groundbreaking examples include the genetic toggle switch^10^ and the “repressilator”^11^).

The roots of this biological circuit metaphor date back to the mechanistic view of the cell; the idea that living systems may be viewed as machines, which, in turn, can be traced as far back as Descartes (and beyond). Characterisations of the cell as a “factory”, a “little engine”, and a “chemical machine” dominated early discourse in cell biology, and the post-war development of cybernetics and computer science, combined with the emergence of molecular biology, provided significant support for this conceptual interpretation of living systems^12^.

The “machine model” of the cell was further cemented by the pioneering work of Jacob and Monod, which established that the *lac* operon^13^ facilitates metabolic switching within *E. coli* in a manner that may be interpreted as a simple Boolean circuit. In his highly influential popular book, *Chance and Necessity* (a chapter of which is titled “Microscopic Cybernetics”), Monod made explicit the connection between biological processes and the basis of electronic circuits:

“The logic of biological regulatory systems abides not by Hegelian laws but, like the workings of computers, by the propositional algebra of George Boole.”^14^

Since the discovery of the *lac* operon, the machine model has largely dominated molecular biology^15^, and the notion of the genome serving as a “genetic program” is still relatively common^16^. Of course, abstracting biological processes to the level of circuits (Boolean or otherwise) or even programs may be a necessary step in terms of making sense of their operation. Conversely, in the context of engineered biological systems, the availability of a set of well-characterised, modular and tunable components are perhaps necessary for creating controllable and predictable systems^17^. We emphasise here that we do not seek to overturn or abandon an extremely powerful and useful metaphor. However, it may give a misleading impression regarding the reliability and predictability of genetic circuits, compared to their electronic counterparts^18^. Moreover, as we argue in this paper, by restricting ourselves to the computational palette offered by (Boolean) combinatorial logic, we inherently (and seriously) limit the power and scope of synthetic biology.

It is perhaps instructive at this point to discuss the field of molecular computing^19^, which many see as a conceptual precursor to synthetic biology, if not a direct predecessor. Although the notion of computing with individual atoms and molecules was attributed to Richard Feynman as early as the 1950s^20^, the field was only reified in 1994, when Leonard Adleman published his groundbreaking paper on computing solutions to the Hamiltonian Circuit Problem using molecular operations with strands of DNA^21^. At the time, a commonly-held belief was that the future promise of DNA computation^22^ lay in solving instances of hard computational problems that were beyond the capabilities of traditional silicon-based computers^23^. Much early work on producing general DNA-based computational models focussed on molecular emulation of the Boolean circuit model^24,25^. Discussion of the field’s overall direction was, for a time, dominated by the quest for the “killer application”, the application of DNA computing that would establish its superiority over existing computational substrates^26,27^. In some ways, this may be viewed as a restricted form of the search for so-called “quantum supremacy” in the quantum computing domain^28^.

As the field of molecular computing evolved, it rapidly became clear that the killer application would not be found by solving large instances of computationally hard problems using any variant of Adleman’s original model. The elegance of the original solution derived from the encoding used to construct all possible paths through a given graph; vertices and edges were represented as strands of DNA that self-assembled into a collection of paths, which was then rapidly filtered in parallel using molecular manipulations to gradually extract valid solutions. Crucially, this reasonable run-time required the entire solution space for a given problem instance to be represented in an initial “test tube” at the start of the computation; the problem was to find the “needle” (valid solution(s)) in a molecular “haystack”. Essentially, the entire space of solutions was tested in polynomial time using molecular operations, but this came at the cost of having to represent the whole solution set in a single volume of liquid^22^. For even small problem instances, the amount of DNA required to represent all possible solutions would be astronomical; Hartmanis calculated that if Adleman’s experiment were scaled up from 7 to 200 vertices, the amount of DNA required would weigh more than the Earth^29^. We note, however, that this inherent time-space tradeoff would appear to hold regardless of whether the underlying substrate is DNA, silicon, or a more exotic material (with the possible exception of substrates for quantum computers^30^).

Importantly, this assessment emphasised the fact that the killer application would probably not be derived from single-use, finite DNA-based systems (albeit ones that were large and potentially massively-parallel in nature). Subsequent influential work on DNA self-assembly^31^ and strand displacement architectures^32^ emulated traditional computational models such as cellular automata^33^ and artificial neurons^34^, but also took advantage of biochemical processes that provided power beyond mere miniaturisation. That is, rather than simply harnessing the potential for massively-parallel laboratory operations on large (but finite) numbers of molecules, these systems co-opted specific physical and chemical properties of the underlying substrate to generate the potential for autonomous operation and dynamic behaviour. In the context of synthetic biology, we suggest the killer application for engineered living systems will be similarly derived; one that will use features of the living system that are unavailable via traditional silicon-based substrates, for the purposes of applications that exploit autonomy and the ability to perform in uncertain environments.

## The search for cellular supremacy

If we accept that a driving motivation for synthetic biology should be the search for a killer application, then this naturally leads to a consideration of the class of problems in which engineered living systems might offer what we call cellular supremacy (deliberately echoing the well-established notion of quantum supremacy^28,35–37^). That is, we seek a set of problem domains in which cell-based systems will offer capabilities beyond the reach of existing computers, due to cost or technological constraints. Simply put, cellular supremacy will be determined by the set of problems that biocomputers can practically solve that traditional microprocessor-based devices cannot. To identify this set of problems, we naturally focus on the “added value” that living systems offer beyond silicon-based hardware. In the following sections, we discuss a number of features of living systems that we believe give synthetic biology-based solutions a distinct “edge” over traditional computers. Importantly, we focus on aspects of computation and information transformation, rather than simply on the ability of the cell to act as a micro-scale drug precursor “production plant” or miniaturised bio-sensor. While these applications are important and valuable, they are not the main focus of the current discussion, which centres on the general computational capabilities of living systems if we go beyond the limitations of Boolean logic-based systems. In what follows, we use the term “cellular computing”^38,39^ to emphasise this focus on computation.

When assessing cellular computing against traditional computing, it may be useful to frame the comparison using a scheme that emerged from an exercise on roadmapping the so-called “unconventional computation” domain. The authors identified a number of criteria or benchmarks against which emerging computational models may be measured, each accompanied by its own motivating question^40^. In what follows, we focus on the quality criterion, which asks the following question: Does the alternative model offer qualitatively different computation to traditional models? The authors define “quality” in terms of precision, richness, stochasticity and repeatability. Quality is concerned with the ability of the system to provide an analysis of inputs that is different to that offered by a traditional computer. If we focus on problems that fall within the remit of traditional silicon-based machines, and use existing metrics, then cellular computing will inevitably fall short on some of the other criteria identified^40^, such as speed and cost. However, we would argue that the real power of cell-based systems derives from the richness of their internal (and collective) architectures, and on their inherent stochasticity.

This leads us to a consideration of the specific computationally expressive mechanisms that are available to us, and which act both inside and between living cells. In what follows we focus on five (non-exclusive) features of living cells that (we believe) offer the most potential in terms of the search for cellular supremacy; these are reconfigurability (that is, the ability to change internal state/structure in response to signals), noise (the ability to not only tolerate but to harness and exploit biological “messiness”), concurrency (the multitude and richness of inter-cellular processes that facilitate massively-parallel communication and coordination), representation (the ability to use non-binary representations for signals), and evolution (the population-level ability to seek out novel solutions to problems over time). By harnessing these capabilities, we will obtain bio-computing systems that are self-organising, self-repairing, resilient, distributed, and adaptive^41^.

First, we consider aspects of computational theory that suggest how cellular computers may out-perform traditional machines. We then describe a number of features of biological systems that lend themselves to non-standard computation. Taken together, these two perspectives point towards a number of possible areas for investigation in which the killer application for cellular computing may reside.

## Information processing beyond digital logic

The current definition of digital computation is based on the abstract model defined by Turing in the 1930s^42^, and the von Neumann architecture^43^ used to implement the types of computations performed by the Turing Machine. Although Turing’s model provides a framework for answering fundamental questions about computation, “…as soon as one leaves the comfort provided by the abundance of mathematical machinery used to describe digital computation, the world seems to be packed with paradoxes.”^44^.

The important thing to note here is that although genetic circuits may appear to behave digitally, it is only the collective behaviour of a large number of inherently analogue components that give rise to this property. The fact remains that we still currently lack any formal framework within which to argue that a cell computes, according to any understood model of computation (although recent work has started to address this^45^).

**Fig. 1:**
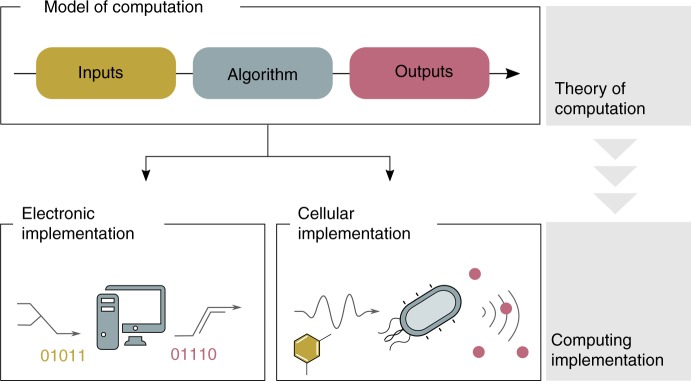
The cell as a “physical” computer. A model of computation formally defines inputs and outputs, as well as how an algorithm processes inputs into outputs. Though the same theoretical model of computation can be physically implemented in many different ways, the nature of computation remains the same. Electronic implementations receive electronic data for inputs/outputs, while cells are able to sense/deliver a wide range of physical, chemical and biological inputs/outputs. The encoding of information into inputs can be done in different ways. Temperature, for instance, can be encoded as the height of mercury in a tube, the voltage of an electronic thermometer or the state of a DNA thermosensor

The nature of computation is not of purely theoretical interest. Mathematical models of computation and their properties inform the engineering of their physical manifestations (Fig. 1). Many such implementations may be possible, but all inherit the characteristics of their abstract counterparts—both their abilities and their limitations. The fact that the nature of computation within a given model is independent of its implementation allows the application of theoretical computer science to all kinds of physical systems, including cells. However, the cost of this generality is a semantic gap between the model and the physical processes that actually perform the computation. That is, mapping computations from an abstract model to a real system may be more or less difficult, depending on the model chosen. The cellular computing substrate is quite different from that of silicon computers, offering opportunities for implementing some models with a narrower semantic gap. Practical considerations such as these could guide future applications of cellular computing.

Conventional silicon computers are fundamentally implementations of a deterministic, centralised and digital model of computation, and they excel at computational tasks which are easily described by such models. In contrast, cellular computing has been optimised over billions of years of evolution to perform very different computational tasks, and we are unlikely to find cellular supremacy in applications such as discrete mathematics, sending emails or reading documents. However, computer science has developed models in which the nature of computation is quite different to that of the Turing Machine^46–53^ and computation in cells appears to share some properties with these less conventional models. With this in mind, the application of theoretical computer science to cellular computation may still present routes to cellular supremacy in those areas where the properties of cellular and traditional computation differ.

Here, we introduce a selection of theoretical aspects of computing that impact significantly upon the nature of computation, all of which seem to be exploited in the cell for processing information, but whose theoretical implications may not be familiar to those outside the field of computer science.

## Computational states for computations over time

Not all models of computation are equally powerful, that is, they are not equivalent in terms of the range of computations they can possibly describe. By this metric, combinatorial logic circuits are extremely weak, since the output of a circuit is purely a function of its current inputs (that is, there is no “memory”), and this severely restricts the range of computations that are possible with a single circuit. Clearly, there are models of computation more powerful than combinatorial logic, that is, models which can describe all computations that are possible with combinatorial logic circuits and more. In this regard, two extremely powerful models of computation, the Lambda calculus^54^ and the Turing Machine^42^ are particularly important. These two models are both equally powerful, but quite different in their formulation—the Lambda calculus is a model of functional computation, while the Turing Machine models stateful computation. Here we focus on stateful computation, as states enable functions that operate over time, such as memory, learning and adaptation.

**Fig. 2:**
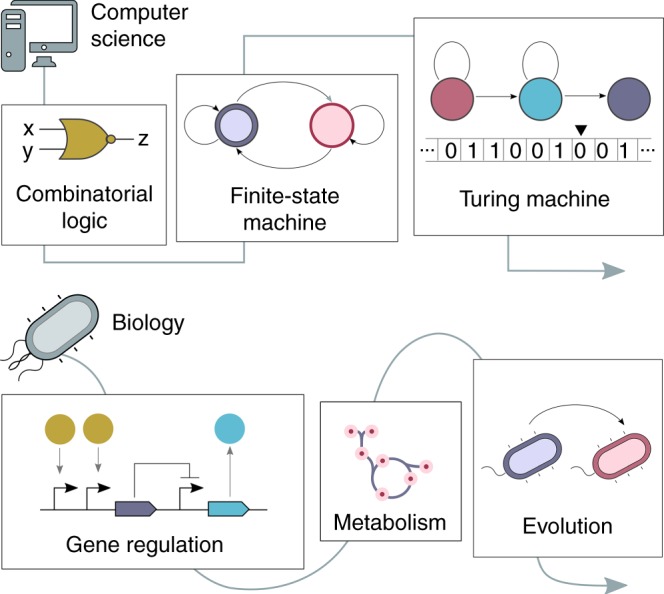
Cells could provide more than logic circuits. Computer science has developed models of computation that are far more powerful than combinatorial logic, such as finite-state machines or the Turing Machine. These models are more powerful in that they allow processing of a wider range of inputs into outputs, and in many more ways, than are admissible by combinatorial logic. Similarly, living systems have evolved a variety of computational processes to allow cells to process information. A simple model, used extensively as the basis for engineering combinatorial logic circuits in cells, is the standard representation of the central dogma (CD) of molecular biology. However, this model does not incorporate core cellular mechanisms such as metabolism, or processes such as evolution, which may provide possibilities for building more powerful, but as yet unknown, models

The output of stateful computations depends not only on the current input, but also on the current state, which encodes information about previous inputs that are no longer present. Models of computation such as finite state machines (FSMs) (shown in Fig. 2) exceed the capabilities of combinatorial logic, and are extremely powerful models of computation—the central processing units of modern digital computers are built from sequential logic circuits, which are implementations of FSMs. Even so, they cannot describe all computations that are available to the Turing Machine model, since they have only a bounded number of states. Turing machines are similar to FSMs, but have an unbounded memory (commonly conceptualized as a tape), which endows them with an unbounded computational state space (number of distinct configurations). Consequently, Turing Machines can compute anything that any real computer can, and in fact, computer science starts from the notion that Turing Machines (TMs) demarcate what is (and is not) computable^42^. From a practical perspective, these considerations of the limits of computability might be of less conceptual value than the strategy that TMs employ to decouple the complexity of the machine from the number of states on which they can operate.

Modern digital computers, as with any real physical system^55^, lack the unbounded state space required for implementing a Turing Machine. However, their state space is so vast as to make them practically indistinguishable from implementations of Turing Machines (consider that even systems with an extremely modest 1 MB of storage have at least $2^{10^6 \!\times\! 8}$ states). It is therefore unlikely that cellular computing will outperform silicon purely on the basis of enabling more complex stateful computations. Nevertheless, synthetic biology has already engineered successful in vivo implementations of stateful computations^10,56,57^, and there also exists significant theoretical work linking stateful models of computation to biological implementations^58,59^. This work is undoubtedly of considerable importance for a broad range of synthetic biology applications, and since biological systems naturally engage in stateful computation^60–62^, statefulness or “memory” will likely be a key component in future engineered biocomputations of any significant complexity.

## Noise permits stochastic and non-deterministic computations

Combinatorial logic circuits, FSMs and TMs model deterministic computations, which are essentially step-wise descriptions of mathematical functions that map inputs to outputs, where each step follows in a predetermined way from the previous step. Deterministic computation can be generalised to include stochastic and non-deterministic computation. Stochastic (or probabilistic, or randomised) computing refers to algorithms which can make random choices during their execution. Specifically, a given input may generate a number of different computational paths, each with some probability, and the cumulative probability of all such paths is equal to one. Probabilistic algorithms are a cornerstone of computer science^63^, and are used to solve approximately, but efficiently, hard optimisation problems for which deterministic algorithms would be intractable. One of the most important algorithms in systems biology, the Gillespie algorithm, is stochastic. Gillespie showed from basic quantum mechanics that a set of biochemical reactions must be understood as a stochastic process^64^, and offered an efficient way for simulating (sampling) its dynamics^65^. Therefore, cells may already be seen as “stochastic processors” that could provide a substrate for implementing probabilistic algorithms. Indeed, the utility of stochastic processors has already been recognised in the silicon world^50,66^.

In non-deterministic models of computation, as in stochastic ones, there may be many computational paths from input to output, but crucially each of these computational paths is explored simultaneously by the algorithm, with a result analogous to parallel execution of multiple distinct deterministic algorithms. While non-determinism has considerable impact on theoretical computer science, practical implementations of non-deterministic computation remain elusive. Nevertheless, algorithmic solutions to some computational tasks can be significantly easier to express as non-deterministic, and biological systems extensively exploit non-determinism, both at the population level^67,68^ and at the scale of evolution^69^. Perhaps more speculatively, we might also consider the role of quantum effects in biology^70^, and whether or not these may be harnessed for the purposes of non-deterministic algorithms in biological systems.

## Distributed computing can exploit concurrency

For some models of computation, the definition of an algorithm is “sequential”, and implies an ordering of the computational steps. Implementations of these models enforce these “sequential semantics”, because sequential execution may be a strict requirement to ensure the correctness of an algorithm. However, for certain computations, algorithms exist in which the ordering of computational steps may be relaxed, or abandoned entirely—in computer science terms, the algorithm displays a level of concurrency. In such situations, if multiple computing devices may interact and coordinate their efforts as a “distributed system”, concurrent parts of an algorithm may be executed in parallel, potentially speeding up computations significantly.

The utility of such distributed computing is not limited to performance advantages. For some problems, sequentiality is desirable, but the nature of the computing substrate makes it impractical (or even impossible) to enforce^71^. For instance, at a fundamental level, and even within a single cell, natural cellular computation is concurrent and distributed. Computation is performed by stochastic biochemical processes—the probabilistic interactions of spatially separated molecular computing “agents”, many of which can happen in any order. Computer science has long used distributed and concurrent models of computation such as actors^49^, Petri nets^51^, process algebras^72^ and population protocols^52^ to describe computations in just these situations.

## Analogue computing allows for continuous non-binary inputs

The nature of computation described so far has been digital; that is, information is represented using a set of discrete values. However, many physically relevant quantities vary continuously. Even the signals that encode binary values in electronic logic circuits are, in reality, stored as continuous-valued voltages. Such signals must be discretised in order to represent digital values. In contrast, analogue computation allows continuous signals to be used directly in the computational steps of an algorithm, and for representation of information as continuous values.

Although many cellular computations involving binary “yes/no” decisions may be interpreted as digital computations, and digital logic computations are certainly suited to applications such as biosensors, cells often exhibit graded responses to stimuli that are more appropriately viewed as analogue computations^62,73^. Furthermore, the biochemical processes responsible for cellular computations involve discrete interactions of discrete molecules, but are also inherently stochastic. Cellular computing may, therefore, be viewed as both digital and stochastic, or as analogue computation with noise^74^, and the viability of the cell as a substrate for synthetic analogue computations has already been demonstrated^75^.

Whilst some models of analogue computation such as the Blum-Shub-Smale machine^53^ or neural networks^46^, use arbitrary precision signals to perform super-Turing computation, it is not clear whether realisations of analogue computation will be able to provide more computational power than digital computation, since physical laws forbid the existence of such an idealised computer^55,76^. However, for digital models of computation, the abstraction of continuous signals into digital represents a semantic gap between the formal definition and implementation of a computation. Models of analogue computation admit continuous signals, which not only narrows this semantic gap, but also allows more powerful computational primitives to be used for defining algorithms^77^. Consequently, depending on the computation and the computing substrate, an analogue algorithm might have a more intuitive implementation, require fewer components to implement, and be considerably more energy-efficient than its digital counterpart^74,75^.

## Engineering complex cellular computing systems

**Fig. 3:**
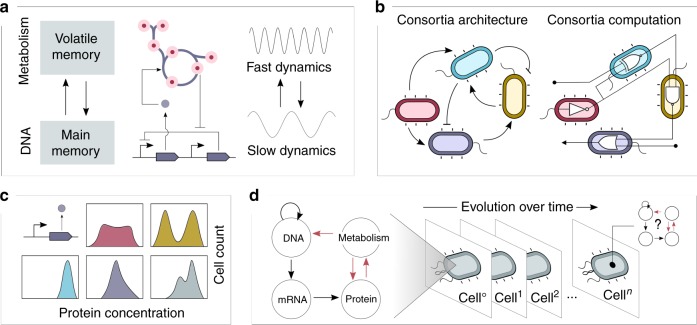
Cellular information-processing fundamentals that go beyond combinatorial logic circuits. **a** Whole-cell computations, merging genetic and metabolic circuits, could achieve more ambitious goals than genetic circuits alone. Cells have evolved intricate networks that make simultaneous use of the varied features of both genetic and metabolic processes. In terms of information storage, metabolism presents a volatile memory, while DNA sequences are able to store information in a more stable fashion. Coordinating the use of different types of memory is a fundamental aspect of complex computer architectures. The dynamic difference is also a potential source of complexity if coupled; metabolic reactions operate on a faster timescale relative to genetic regulatory networks. **b** Multicellular computing (right) is currently implemented by connecting the output of one strain to the input of another. Social interactions among cells (left), such as cooperation, mutualism, competition or commensalism, are not considered in general. However, social interactions are fundamental in natural communities—they provide stable architectures executing a desired computation. **c** Gene expression noise is intrinsic to living systems; the panel figure shows different patterns for gene expression. Despite the fact that all are described as being on, there are different types of expression—thus different on/off standards. **d** The cell as a general-purpose machine. As the basis for a model of computation, the central dogma of molecular biology can be expanded to include metabolism. Evolutionary processes may also be included as major forces guiding information-processing in cells, since they allow the purpose of cellular computations to adapt over time

There are certainly deep physical connections between chemistry and electronics^78^, but the fact remains that the cellular environment is a radically different computing substrate than silicon. Although this difference might make cells unsuitable for computational tasks traditionally dominated by conventional computers, it could also offer opportunities to explore more unconventional models of computation. Aside from gene regulation, which has been useful for engineering biological logic circuits, a number of processes and features exist in natural systems which may offer computational capabilities. Here, we identify four such resources as promising in terms of their information-processing capabilities (Fig. 3).

## Towards whole-cell computation

Synthetic cellular computing systems have generally focussed on DNA components that are isolated from the many other processes within a cell^79,80^. However, evolution has shaped intricate information-processing networks whose function emerges from the interplay between many layers of the cellular machinery^81^. For example, bacteria adapt to nutrient fluctuations by sensing extracellular cues, transducing such signals to the genetic machinery, and ultimately adapting their metabolic state to make maximal use of carbon sources. This requires a careful whole-cell orchestration of trafficking processes at the levels of membrane, cellular signalling pathways, gene expression programmes and metabolic activity.

In a similar fashion, synthetic systems that exploit whole-cell interactions offer exciting opportunities to expand the range of computational tasks that can be achieved with living systems. Metabolic circuits, in particular, produce analogue dynamics that could allow us to move beyond basic Boolean operations^82^. Circuits integrating genetic programs with metabolic activity have been successfully engineered to improve the productivity of microbial cell factories^83–85^, but their potential goes far beyond simple production. Notably, retrobiosynthesis approaches based on generic representations of reactions, known as reaction rules, expand the design space of metabolic circuits by predicting novel synthetic pathways connecting metabolism and gene expression^86–88^. Machine-learning is increasingly being applied by such retrosynthesis algorithms to select the best candidate reactions in the search through the chemical space^89,90^.

Moreover, experimental and theoretical work has shown that gene expression noise can permeate to metabolism^68,91^, suggesting the possibility of processing information using metabolic heterogeneity. The interplay between gene expression and metabolism can be conceptualised in terms of memory systems employed in most computer architectures. These have a long-term static memory, conceptually similar to information stored in DNA, and a short-term volatile memory, akin to the fast dynamics of metabolism (Fig. 3a). Computer science has developed efficient techniques for managing data flows across these two memories to exploit their characteristic timescales. There is some evidence that cells also exploit such differing timescales to their benefit^62^, and, ultimately, the ability to encode signals in different frequency domains may allow us to move from classic Boolean abstractions to more complex coding systems.

Whole-cell computation presents major challenges because, as the size of synthetic circuit grows, so does their footprint on the cellular host. Synthetic circuits do not work in isolation, but continuously draw resources away from vital functions of the host^92^. These resources include energy, polymerases for transcription, ribosomes for translation, enzymatic co-factors, and so on, the depletion of which has a negative impact on native processes. This is commonly known as the cellular burden, and has gathered substantial attention in the community^93^. Recent work has aimed at identifying relevant sources of burden imposed by synthetic constructs^94,95^. Together with increasingly powerful mathematical models for cell physiology^96,97^, these experimental efforts are paving the way for accurate prediction^98^ and control of burden^99^, which will enable the construction of complex circuitry that takes full advantage of the cellular machinery^100^.

## The power of multicellular consortia

The burden imposed by synthetic biological constructs places a fundamental ceiling on the information processing capacity of a single cell. In order to execute larger and more complex computations, living systems harness the capabilities of multicellular ensembles, such as the vast neural networks in the brain. Distributing computational demands across a population of specialised cells lowers the burden on each individual cell, and offers a way to more easily scale the complexity of the computations being performed. Such scalability is uniquely fostered in biological systems by the inherent ability of living cells to self-replicate. Similar distributed approaches have been employed in engineered biological systems, where an algorithm is spread across a consortium of different microbes that are able to communicate and interact (Fig. 3b). Many theoretical^101–105^ and experimental^106–112^ examples demonstrate the benefits of distributing computation in this way, as well as a growing number of tools for their simulation^113–120^ and assembly in living cells^121,122^.

Although the development of engineered multicellular consortia is possible with current technologies, their structure is often fragile, and their information-processing capabilities are dwarfed by microbial communities found in nature^123^. A key difference in naturally-occurring systems is that extensive resources and diverse mechanisms are used to establish and coordinate the complex social behaviours of a communityʼs participants^123^. Social relationships such as cooperation, mutualism, competition and commensalism are used concurrently to drive a required community structure that can potentially change over time in response to the environment. Advances in our ability to control cell-to-cell interactions, either through spatial patterning of cell populations^112^ and/or orthogonal communication channels^121,124^, will be crucial to future developments in this area.

A feature of multicellular consortia that pushes us beyond the capabilities of classical electronic computers is their highly flexible and re-configurable nature^125^. In computer science, a computer architecture describes the structural relationship between different components of a system (e.g. the processor and memory) and constrains how algorithms run on the system. A program compiled for one type of computer architecture may not be executable by another due to these physical differences. The architecture of most electronic computers is fixed during a computation and has been honed to perform primitive digital operations in quick succession, guided by a set of instructions (the code). While this architecture is general enough for tackling many types of task, its static architecture makes it sub-optimal for most of them. In a cellular consortium, such a severe trade-off between flexibility and optimality (e.g. in regards to energy consumption or speed) need not exist, as the cellular community can dynamically change its physical composition and the interactions present to produce a “living architecture” that better matches the problem at hand. Furthermore, such reconfiguration is possible even during a computation itself, offering a level of control and flexibility that electronic computers are unable to match. Harnessing these unique capabilities will offer synthetic biologists new approaches to solving problems and require us to rethink what a computer architecture can be^126^.

## Encoding signals using gene expression noise

Combinatorial logic, which relies on well separated on/off signals to encode information, constitutes a valuable framework with which to understand and build genetic circuits. Even if molecular signals (for example, a transcription factor that down-regulates a promoter) do not have absolute and constant values, the notion of a promoter being repressed or not (thus being binary) is intuitive. Nevertheless, the use of Boolean logic for engineering cellular circuits is often challenged by the fact that molecular signals are intrinsically noisy, and therefore stochastic. Each signal will have different on/off values (Fig. 3c), and not all are compatible, which makes building a large combinatorial circuit a significant challenge^127^. Combinatorial logic is, therefore, a good example of how a given theoretical model of computation may be easily implemented with one physical toolkit (electronic), but not with another (molecular).

The voltage that runs through electronic wires is also noisy, but computer engineers overcame this problem by arranging for thresholds at the input and output of logic gates, so that analogue values could be abstracted into two unique values: 0 and 1. In contrast to this, living systems have evolved to benefit from signal variability. Stochasticity has been shown to be useful in coordinating the expression of large sets of genes^128^, in producing the phenotypic variability that allows for bet-hedging strategies that promote survival in fluctuating environments^67,129^, and as a key component of evolution under tight selection criteria^69^. Therefore, models of computation incorporating stochasticity would seem to be necessary to best fit the intrinsic properties of living systems.

Unlike silicon computing devices, where noise is a consequence of random signal fluctuations, cellular systems are capable of encoding important information into noise patterns. For example, the change in gene expression noise according to intracellular physical distances^130^ reveals information about the structural architecture of the cell. Also, noisy patterns in the cellular machinery of *Staphylococcus aureus* (that lead to different infectious cell types) originate in upstream molecular events^131^. These examples, among others^75^, highlight the fact that the shape of non-digital biological signals is used to effectively transmit information. Therefore, the challenge is to use noise for implementing computations — an area where cellular computers clearly outperform conventional ones. In the meantime, current efforts in the discretisation^132^, stabilisation^133,134^ and reusability^135^ of noisy signals may help to understand, and harness, the dynamic complexity of molecular interactions.

## Evolution as an information-processing mechanism

Evolutionary computing is a field within computer science that develops and applies algorithms inspired by Darwinian evolution^136^. Different implementations of this idea exist, such as genetic algorithms, evolutionary strategies or genetic programming, but the underlying principles are similar: a set of candidate solutions to a problem (an initial population) compete under some pre-defined fitness criteria, and the fittest individuals are selected to form the next generation (via crossover and/or mutation). An important advantage of these techniques is that they allow for the dynamic optimisation of solutions throughout potentially vast search spaces.

Both computer scientists and living systems use evolutionary algorithms to generate algorithmic solutions. Evolutionary processes have also been used to design biomolecules^137,138^, libraries of biological components^139^ and even to evolve non-functional genetic circuits into functional ones^140^. Despite this, evolutionary processes are often omitted when engineering computing circuits in living cells. Harnessing the power of such information-processing mechanisms for predefined functions seems to be difficult, and the rational engineering of autonomous evolutionary computing in living cells is still an overarching challenge.

In order to start addressing this challenge, it will be important to formalise the effects of evolutionary dynamics on the flow of genetic information. A potential initial step would be to expand on the standard representation of the central dogma (CD) of molecular biology to include evolutionary dynamics, in an attempt to describe the model of computation of a living cell (Fig. 3d). Expansion of the CD-based model is not a new idea; for example, the inclusion of metabolic processes has previously been suggested^141^, since, as evidenced above in “Towards whole-cell computation”, metabolism is a crucial omission from the point of view of information processing. A complete model of the flow of genetic information at a given point in time needs to include both genetic and metabolic processes, and dynamic models of computation inside living cells should include evolutionary processes that operate on genetic information.

Theoretical models of computation may find valuable and perhaps novel implementations within this dynamic representation of the CD. Recent developments in optogenetics are promising to this end, since these show how genetic elements dynamically shape their response in relation to external signals^142^. Importantly, these studies linking environmental signals to intracellular responses in a predictable way, as well as directed evolution efforts^143^, are undertaken in tightly controlled systems. However, the mechanisms that allow a cell to thrive in challenging and dynamic environments are largely unpredictable. For problems like bio-remediation which intrinsically involve ever-changing environments, natural cellular computers clearly show superiority compared to conventional ones.

## Applications of cellular computing systems

As already discussed, cellular computing is unlikely to compete with conventional computers in domains for which the latter are specifically engineered. Although future biological systems may offer competitive performance in related areas such as data storage^144^, the benefits are largely limited to the exploitation of material factors such as miniaturisation and longevity of components.

The identification of promising applications for cellular computing has been largely guided by the observation that living systems operate best in application domains that are not easily reachable by conventional computers. Successes in bio-remediation^145^, bio-production^146^ and targeted therapeutics^147^ indicate that this is indeed a fruitful line of inquiry. Nevertheless, the implementation of conventional computations with unconventional computing substrates does not, in and of itself, constitute cellular supremacy. Rather, supremacy must be derived from the fact that the type of computation performed by a conventional computer is qualitatively different to that executed by living systems. Current implementations of computations within living cells are generally based on combinatorial or sequential logic circuits mapped onto equivalent genetic circuits; while these are useful metaphors for both understanding and engineering living systems, digital logic is not easily implemented using a cellular substrate. Furthermore, logic circuits offer a relatively bland computational palette compared to the richness of biology. For this reason, future developments in cellular computing should focus on models of computation that both accommodate and exploit the natural abilities of the cell, and avoid forcing biological systems into artificial (and often unsuitable) architectures^148^.

This does not mean that cellular computing must necessarily “reinvent the wheel” when it comes to models of computation. Living systems share many characteristics with in vitro platforms for computation, and as well as the considerable body of theoretical work concerning molecular computing, cellular computing may draw on cell-free systems as a practical resource for faster prototyping with a greater degree of control^149–151^. Furthermore, the relationship between computer science and natural computing is often synergistic. As described earlier, models of computation that transcend logic circuits are available which have yet to be explored by cellular computing to any great extent. In return, biological systems offer computational features that are (and are expected to remain) unavailable to silicon implementations of existing computational models. It is at this intersection that exciting opportunities for cellular supremacy emerge. For example, in the past decade it has become increasingly clear that a number of biological processes show quantum mechanical properties^152,153^. In particular, there is strong experimental evidence that long-lived quantum coherence is involved in photosynthesis^154^, and that quantum tunnelling is active in enzyme catalysis^155^. Since most quantum computing devices are built and run under stringent environmental conditions (at temperatures approaching absolute zero), the opportunity to control quantum effects in a biological system that “runs” at room temperature, through the emergence of a “quantum synthetic biology”, could turn out to be a game changer in the quantum supremacy race. More to the point, realising models of quantum computation^156^ using quantum biology could yield a cellular computer capable of a radically different kind of computation than silicon^153^.

Although we have, until now, focussed primarily on bacterial cells, there does, of course, exist a multiplicity of cell types that possess rich and complex computational capabilities. For example, plants can respond to environmental signals and stresses in a way that is reminiscent of signal processing in neural networks (the so-called “plant perceptron”)^157^. Cultured neuronal cells have been used to control flight simulators^158^ and robots^159^, and slime mould has a rich repertoire of spatial computing capabilities^160^. Additionally, we do not dismiss the possibility of “hybrid” architectures, in which cellular systems are interfaced to more traditional, silicon-based machines. Such connections may allow for subtle and sensitive adjustments to cell state, for example^161^, but these hybrid systems will still fundamentally rely on the properties of the living material.

As we have already argued, natural cellular computing operates at vast scales, in a distributed manner, and in the presence of considerable noise. Consequently, biological metaphors have served as inspirations for models of amorphous computation, for which cellular computing is a promising implementation technology^162^, especially at scales inaccessible to silicon. Robotics has also drawn inspiration from biological computation, particularly in relation to morphological computing, which takes advantage of the physical properties of computing agents in order to achieve more efficient computations^163^. By using intrinsic physical properties of the computational substrate to “outsource” parts of the computation, increasingly complex computations can be carried out whilst maintaining relatively simplistic control structures^164^. Living systems are an ideal implementation technology for morphological computation, since they not only compute solutions to individual instances of problems, but continuously compute and adapt in order to embody an efficient general solution. As we have previously argued, conventional silicon computers have inflexible architectures by comparison, which must sacrifice efficiency for generality. These qualitative differences between cellular and conventional computing suggest that applications such as terraforming and smart material production may remain beyond the reach of silicon computers, but in contrast, strategies for both applications based on living technologies have already been proposed^165,166^.

In this perspective, we have championed the notion of cellular supremacy in an attempt to focus attention on the high-impact areas in which synthetic biology can have a truly transformational effect. We call on the community to consider cellular supremacy as a framing device for future work, and to explore in a systematic fashion how it may be established. By accepting the idea of cellular supremacy, we naturally acknowledge the richness and power of living systems. And by ceding a degree of control to biology, we may yet open up a much wider range of applications and perspectives on information processing in nature.

## References

[1] Andrianantoandro, E., Basu, S., Karig, D. K. & Weiss, R. Synthetic biology: new engineering rules for an emerging discipline. *Mol. Syst. Biol*. **2**, 2006.0028 (2006). 10.1038/msb4100073 PMC1681505 16738572

[2] Ausländer S, Ausländer D, Fussenegger M (2017). Synthetic biology—the synthesis of biology. Angew. Chem. Int. Ed..

[3] Amos, M. & Goñi-Moreno, A. Cellular computing and synthetic biology. In *Computational Matter* (eds Stepney, S., Rasmussen, S. & Amos, M.) 93–110 (Springer, 2018).

[4] Chubukov V, Mukhopadhyay A, Petzold CJ, Keasling JD, Martín HG (2016). Synthetic and systems biology for microbial production of commodity chemicals. npj Syst. Biol. Appl..

[5] de Lorenzo V (2018). The power of synthetic biology for bioproduction, remediation and pollution control: The UN’s Sustainable Development Goals will inevitably require the application of molecular biology and biotechnology on a global scale. EMBO Rep..

[6] Slomovic S, Pardee K, Collins JJ (2015). Synthetic biology devices for in vitro and in vivo diagnostics. Proc. Natl. Acad. Sci..

[7] Bray, D. *Wetware: A Computer in Every Living Cell* (Yale University Press, 2009).

[8] Clarke LJ (2019). Synthetic biology – pathways to commercialisation. Eng. Biol..

[9] Condon A (2018). Will biologists become computer scientists?. EMBO Rep..

[10] Gardner TS, Cantor CR, Collins JJ (2000). Construction of a genetic toggle switch in *Escherichia coli.*. Nature.

[11] Elowitz MB, Leibler S (2000). A synthetic oscillatory network of transcriptional regulators. Nature.

[12] Nicholson DJ (2019). Is the cell really a machine?. J. Theor. Biol..

[13] Jacob F, Monod J (1961). Genetic regulatory mechanisms in the synthesis of proteins. J. Mol. Biol..

[14] Monod, J. *Chance and Necessity: An Essay on the Natural Philosophy of Modern Biology* (Alfred A. Knopf, 1971).

[15] Alberts B (1998). The cell as a collection of protein machines: Preparing the next generation of molecular biologists. Cell.

[16] Ronald J (2014). Planer. Replacement of the “genetic program” program. Biol. Philos..

[17] Wang B, Kitney RI, Joly N, Buck M (2011). Engineering modular and orthogonal genetic logic gates for robust digital-like synthetic biology. Nat. Commun..

[18] de Lorenzo V (2011). Beware of metaphors: Chasses and orthogonality in synthetic biology. Bioengineered Bugs.

[19] Conrad, M. Molecular computing. In *Advances In Computers*, vol 31, 235–324 (Elsevier, 1990).

[20] Richard, F. There’s plenty of room at the bottom. In *Feynman and Computation*, 63–76 (CRC Press, 2018).

[21] Adleman LM (1994). Molecular computation of solutions to combinatorial problems. Science.

[22] Amos, M. *Theoretical and Experimental DNA Computation* (Springer, 2005).

[23] Lipton RJ (1995). Using DNA to solve NP-complete problems. Science.

[24] Ogihara, M. & Ray, A. Simulating Boolean circuits on a DNA computer. *Algorithmica* **25**, 239–250 (1999).

[25] Amos, M., Dunne, P. E. & Gibbons, A. DNA simulation of Boolean circuits. In *Proceedings of 3rd Annual Genetic Programming Conference*, 679–683 (1997).

[26] Rubin H (1996). Looking for the DNA killer app. Nat. Struct. Biol..

[27] Landweber, L. F. & Lipton, R. J. DNA^2^ DNA computations: A potential “killer app”? In *International Colloquium on Automata, Languages, and Programming*, 56–64 (Springer, 1997).

[28] Harrow AW, Montanaro A (2017). Quantum computational supremacy. Nature.

[29] Hartmanis J (1995). On the weight of computations. Bull. Eur. Assoc. Theor. Computer Sci..

[30] Preskill J (2018). Quantum Computing in the NISQ era and beyond. Quantum.

[31] Rothemund PW (2006). Folding DNA to create nanoscale shapes and patterns. Nature.

[32] Qian L, Winfree E (2011). Scaling up digital circuit computation with DNA strand displacement cascades. Science.

[33] Rothemund PWK, Papadakis N, Winfree E (2004). Algorithmic self-assembly of DNA Sierpinski triangles. PLoS Biol.,.

[34] Qian L, Winfree E, Bruck J (2011). Neural network computation with DNA strand displacement cascades. Nature.

[35] Preskill, J. Quantum computing and the entanglement frontier. *arXiv preprint arXiv*:*1203.5813*, 2012. https://arxiv.org/abs/1203.5813. ***First usage of the term “quantum supremacy” to denote problems that can be solved using quantum computers in a way that significantly out-performs classical machines***.

[36] Boixo S (2018). Characterizing quantum supremacy in near-term devices. Nat. Phys..

[37] Neill C, Roushan P (2018). A blueprint for demonstrating quantum supremacy with superconducting qubits. Science.

[38] Amos, M. (ed.) *Cellular Computing* (Oxford University Press, 2004).

[39] Teuscher, C. Cellular computing. In *Computational Complexity: Theory, Techniques, and Applications*, 465–478 (Springer, 2012).

[40] Stepney, S. & Hickinbotham, S. J. In *Computational Matter* (eds Stepney, S., Rasmussen, S. & Amos, M.) (Springer, 2018).

[41] Amos M, Dittrich P, McCaskill J, Rasmussen S (2011). Biological and chemical information technologies. Procedia Computer Sci..

[42] Turing AM (1937). On computable numbers, with an application to the Entscheidungsproblem. Proc. Lond. Math. Soc..

[43] Von Neumann J (1993). First draft of a report on the EDVAC. IEEE Ann. Hist. Comput..

[44] Konkoli, Z. et al. Philosophy of computation. In *Computational Matter* (eds Stepney, S., Rasmussen, S. & Amos, M.), 153–184 (Springer, 2018).

[45] Horsman, D., Kendon, V., Stepney, S. & Young, J. P.W. Abstraction and representation in living organisms: when does a biological system compute? In *Representation and Reality in Humans, Other Living Organisms and Intelligent Machines*, 91–116 (Springer, 2017).

[46] Siegelmann HT, Sontag ED (1994). Analog computation via neural networks. Theor. Computer Sci..

[47] MacLennan BJ (2004). Natural computation and non-Turing models of computation. Theor. Computer Sci..

[48] Shannon CE (1941). Mathematical Theory of the Differential. Analyzer. J. Math. Phys..

[49] Agha, G. ACTORS: A model of concurrent computation in distributed systems (1986).

[50] Alaghi, A. & Hayes, J. P. On the functions realized by stochastic computing circuits. In *Proceedings of the 25th Edition on Great Lakes Symposium on VLSI*, GLSVLSI ’15, 331–336, (ACM, New York, NY, USA, 2015). ACM.

[51] Peterson JL (1977). Petri Nets. ACM Computing Surveys.

[52] Angluin D, Aspnes J, Diamadi Z, Fischer MJ, Peralta R (2006). Computation in networks of passively mobile finite-state sensors. Distrib. Comput..

[53] Blum, L., Shub, M. & Smale, S. On a theory of computation over the real numbers; NP completeness, recursive functions and universal machines. In *Proceedings of the 29th Annual Symposium on Foundations of Computer Science*, 387–397 (1988).

[54] Church A (1936). An unsolvable problem of elementary number theory. Am. J. Math..

[55] Bekenstein JD (1981). Universal upper bound on the entropy-to-energy ratio for bounded systems. Phys. Rev. D..

[56] Friedland AE (2009). Synthetic gene networks that count. Science.

[57] Lou C (2010). Synthesizing a novel genetic sequential logic circuit: a push-on push-off switch. Mol. Syst. Biol..

[58] Oishi K, Klavins E (2014). Framework for engineering finite state machines in gene regulatory networks. ACS Synth. Biol..

[59] Soloveichik D, Cook M, Winfree E, Bruck J (2008). Computation with finite stochastic chemical reaction networks. Nat. Comput..

[60] Lambert, G. & Kussell, E. Memory and fitness optimization of bacteria under fluctuating environments. *PLoS Genet.* **10**, e1004556 (2014). 10.1371/journal.pgen.1004556 PMC4177670 25255314

[61] Hoffer SM, Westerhoff HV, Hellingwerf KJ, Postma PW, Tommassen J (2001). Autoamplification of a two-component regulatory system results in “learning” behavior. J. Bacteriol..

[62] Vladimirov N, Sourjik V (2009). Chemotaxis: How bacteria use memory. Biol. Chem..

[63] Motwani, R. & Raghavan, P. *Randomized Algorithms* (Cambridge University Press, 1995).

[64] Gillespie DT (1992). A rigorous derivation of the chemical master equation. Phys. A: Stat. Mech. its Appl..

[65] Gillespie DT (1977). Exact stochastic simulation of coupled chemical reactions. J. Phys. Chem..

[66] Alaghi A, Hayes JP (2013). Survey of stochastic computing. ACM Trans. Embedded Comput. Syst..

[67] Ana Solopova J (2014). Bet-hedging during bacterial diauxic shift. Proc. Natl Acad. Sci. USA.

[68] Mona K (2019). A Oyarzún. Stochastic modelling reveals mechanisms of metabolic heterogeneity. Commun. Biol..

[69] Eldar A, Elowitz MB (2010). Functional roles for noise in genetic circuits. Nature.

[70] Marais A (2018). The future of quantum biology. J. R. Soc. Interface.

[71] Lamport L (1978). Time, clocks, and the ordering of events in a distributed system. Commun. ACM.

[72] Ciocchetta F, Hillston J (2009). Bio-PEPA: A framework for the modelling and analysis of biological systems. Theor. Computer Sci..

[73] Scialdone A (2013). *Arabidopsis* plants perform arithmetic division to prevent starvation at night. eLife.

[74] Sarpeshkar, R. Analog synthetic biology. *Philosophical transactions. Series A, Mathematical, physical, and Engineering sciences*, 372, 2014. 10.1098/rsta.2013.0110 PMC3928905 24567476

[75] Daniel R, Rubens JR, Sarpeshkar R, Lu TK (2013). Synthetic analog computation in living cells. Nature.

[76] Heisenberg W (1927). Über den anschaulichen Inhalt der quantentheoretischen Kinematik und Mechanik. Z. f.ür. Phys..

[77] Sarpeshkar R (1998). Analog versus digital: Extrapolating from electronics to neurobiology. Neural Comput..

[78] Woo SS, Kim J, Sarpeshkar R (2018). A digitally programmable cytomorphic chip for simulation of arbitrary biochemical reaction networks. IEEE Trans. Biomed. Circuits Syst..

[79] Goñi-Moreno, A. & Nikel, P. I. High-performance biocomputing in synthetic biology—integrated transcriptional and metabolic circuits. *Front. Bioeng. Biotechnol.* **7**, 40 (2019). 10.3389/fbioe.2019.00040 PMC6421265 30915329

[80] Oyarzún, D. A. & Stan, G.-B. V. Synthetic gene circuits for metabolic control: Design trade-offs and constraints. *J. R. Soc. Interface*, **10**, 20120671 (2013). 10.1098/rsif.2012.0671 PMC3565798 23054953

[81] Chavarría M, Goñi-Moreno Á, de Lorenzo V, Nikel PI (2016). A metabolic widget adjusts the phosphoenolpyruvate-dependent fructose influx in *Pseudomonas putida*. mSystems.

[82] Pandi A (2019). Metabolic perceptrons for neural computing in biological systems. Nat. Commun..

[83] Zhang F, Carothers JM, Keasling JD (2012). Design of a dynamic sensor-regulator system for production of chemicals and fuels derived from fatty acids. Nat. Biotechnol..

[84] Xu P, Li L, Zhang F, Stephanopoulos G, Koffas M (2014). Improving fatty acids production by engineering dynamic pathway regulation and metabolic control. Proc. Natl Acad. Sci. USA.

[85] Liu D, Mannan AA, Han Y, Oyarzún DA, Zhang F (2018). Dynamic metabolic control: Towards precision engineering of metabolism. J. Ind. Microbiol. Biotechnol..

[86] Delépine B, Libis V, Carbonell P, Faulon J-L (2016). SensiPath: Computer-aided design of sensing-enabling metabolic pathways. Nucleic Acids Res..

[87] Delépine B, Duigou T, Carbonell P, Faulon J-L (2018). RetroPath2.0: a retrosynthesis workflow for metabolic engineers. Metab. Eng..

[88] Lin G-M, Warden-Rothman R, Voigt CA (2019). Retrosynthetic design of metabolic pathways to chemicals not found in nature. Curr. Opin. Syst. Biol..

[89] Segler MHS, Preuss M, Waller MP (2018). Planning chemical syntheses with deep neural networks and symbolic AI. Nature.

[90] Liu B (2017). Retrosynthetic reaction prediction using neural sequence-to-sequence models. *ACS Central*. Science.

[91] Kotte O, Volkmer B, Radzikowski JL, Heinemann M (2014). Phenotypic bistability in *Escherichia coli*’s central carbon metabolism. Mol. Syst. Biol..

[92] Nikolados E-M, Weiße AY, Ceroni F, Oyarzún DA (2019). Growth defects and loss-of-function in synthetic gene circuits. ACS Synth. Biol..

[93] Cardinale S, Arkin AP (2012). Contextualizing context for synthetic biology - identifying causes of failure of synthetic biological systems. Biotechnol. J..

[94] Ceroni F, Algar R, Stan G-B, Ellis T (2015). Quantifying cellular capacity identifies gene expression designs with reduced burden. Nat. Methods.

[95] Gyorgy A (2015). Isocost lines describe the cellular economy of genetic circuits. Biophysical J..

[96] Gorochowski TE, Avcilar-Kucukgoze I, Bovenberg RAL, Roubos JA, Ignatova Z (2016). A minimal model of ribosome allocation dynamics captures trade-offs in expression between endogenous and synthetic genes. ACS Synth. Biol..

[97] Weiße AY, Oyarzún DA, Danos V, Swain PS (2015). Mechanistic links between cellular trade-offs, gene expression, and growth. Proc. Natl Acad. Sci. USA.

[98] Borkowski, O. et al. Cell-free prediction of protein expression costs for growing cells. *Nat. Commu.**9*, 1457 (2018). 10.1038/s41467-018-03970-x PMC5899134 29654285

[99] Francesca Ceroni A (2018). Burden-driven feedback control of gene expression. Nat. Methods.

[100] Ceroni F, Blount BA, Ellis T (2016). Sensing the right time to be productive. Cell Syst..

[101] Macia J, Vidiella B, Solé RV (2017). Synthetic associative learning in engineered multicellular consortia. J. R. Soc. Interface.

[102] Sardanyés J, Bonforti A, Conde N, Solé R, Macia J (2015). Computational implementation of a tunable multicellular memory circuit for engineered eukaryotic consortia. Front. Physiol..

[103] Macia J, Sole R (2014). How to make a synthetic multicellular computer. PLoS One.

[104] Goñi-Moreno A, Redondo-Nieto M, Arroyo F, Castellanos J (2011). Biocircuit design through engineering bacterial logic gates. Nat. Comput..

[105] Goñi-Moreno A, Amos M, de la Cruz F (2013). Multicellular computing using conjugation for wiring. PLoS One.

[106] Chen Y, Kim JK, Hirning AJ, Josic K, Bennett MR (2015). Emergent genetic oscillations in a synthetic microbial consortium. Science.

[107] Fiore G (2017). *In-silico* analysis and implementation of a multicellular feedback control strategy in a synthetic bacterial consortium. ACS Synth. Biol..

[108] Urrios A (2016). A synthetic multicellular memory device. ACS Synth. Biol..

[109] Regot S (2011). Distributed biological computation with multicellular engineered networks. Nature.

[110] Danino T, Mondragón-Palomino O, Tsimring L, Hasty J (2010). A synchronized quorum of genetic clocks. Nature.

[111] Tabor JJ (2009). A synthetic genetic edge detection program. Cell.

[112] Tamsir A, Tabor JJ, Voigt CA (2011). Robust multicellular computing using genetically encoded NOR gates and chemical ‘wires’. Nature.

[113] Gorochowski TE (2016). Agent-based modelling in synthetic biology. Essays Biochem..

[114] Rudge TJ, Steiner PJ, Phillips A, Haseloff J (2012). Computational modeling of synthetic microbial biofilms. *ACS Synthetic*. Biology.

[115] Jang SS, Oishi KT, Egbert RG, Klavins E (2012). Specification and simulation of synthetic multicelled behaviors. *ACS Synthetic*. Biology.

[116] Gorochowski TE (2012). BSim: An agent-based tool for modeling bacterial populations in systems and synthetic biology. PLoS ONE.

[117] Goni-Moreno, A. & Amos, M. DiSCUS: A simulation platform for conjugation computing. In *International Conference on Unconventional Computation and Natural Computation*, 181–191 (Springer, 2015).

[118] Naylor J (2017). Simbiotics: A multiscale integrative platform for 3D modeling of bacterial populations. ACS Synth. Biol..

[119] Montagna, S. & Viroli, M. A computational framework for modelling multicellular biochemistry. In *2009 IEEE Congress on Evolutionary Computation*, 2233–2240 (2009).

[120] Kang S, Kahan S, McDermott J, Flann N, Shmulevich I (2014). Biocellion: accelerating computer simulation of multicellular biological system models. Bioinformatics.

[121] Kylilis N, Tuza ZA, Stan G-B, Polizzi KM (2018). Tools for engineering coordinated system behaviour in synthetic microbial consortia. Nat. Commun..

[122] Ji W (2013). A formalized design process for bacterial consortia that perform logic computing. PLoS One.

[123] McCarty NS, Ledesma-Amaro R (2019). Synthetic biology tools to engineer microbial communities for biotechnology. Trends Biotechnol..

[124] Scott SR, Hasty J (2016). Quorum sensing communication modules for microbial consortia. ACS Synth. Biol..

[125] Amos M (2014). Population-based microbial computing: a third wave of synthetic biology?. Int. J. Gen. Syst..

[126] Brenner K, You L, Arnold FH (2008). Engineering microbial consortia: a new frontier in synthetic biology. Trends Biotechnol..

[127] Nielsen AAK (2016). Genetic circuit design automation. Science,.

[128] Cai L, Dalal CK, Elowitz MB (2008). Frequency-modulated nuclear localization bursts coordinate gene regulation. Nature.

[129] Salek MM, Carrara F, Fernandez V, Guasto JS, Stocker R (2019). Bacterial chemotaxis in a microfluidic T-maze reveals strong phenotypic heterogeneity in chemotactic sensitivity. Nat. Commun..

[130] Goñi-Moreno Á, Benedetti I, Kim J, de Lorenzo V (2017). Deconvolution of gene expression noise into spatial dynamics of transcription factor– promoter interplay. ACS Synth. Biol..

[131] García-Betancur J-C (2017). Cell differentiation defines acute and chronic infection cell types in *Staphylococcus aureus*. Elife.

[132] Lu TK, Khalil AS, Collins JJ (2009). Next-generation synthetic gene networks. Nat. Biotechnol..

[133] Tomazou M, Stan G-B (2018). Portable gene expression guaranteed. Nat. Biotechnol..

[134] Aoki SK, Lillacci G, Gupta A, Baumschlager A, Schweingruber D (2019). A universal biomolecular integral feedback controller for robust perfect adaptation. Nature.

[135] Goñi-Moreno A, Amos M (2012). A reconfigurable NAND/NOR genetic logic gate. BMC Syst. Biol..

[136] Eiben, A. E. et al. *Introduction to Evolutionary Computing*, vol 53 (Springer, 2003).

[137] Esvelt KM, Carlson JC, Liu DR (2011). A system for the continuous directed evolution of biomolecules. Nature.

[138] Zhao H, Giver L, Shao Z, Affholter JA, Arnold FH (1998). Molecular evolution by staggered extension process (StEP) *in vitro* recombination. Nat. Biotechnol..

[139] Brödel AK, Jaramillo A, Isalan M (2016). Engineering orthogonal dual transcription factors for multi-input synthetic promoters. Nat. Commun..

[140] Yokobayashi Y, Weiss R, Arnold FH (2002). Directed evolution of a genetic circuit. Proc. Natl. Acad. Sci. USA.

[141] de Lorenzo V (2014). From the selfish gene to selfish metabolism: revisiting the central dogma. Bioessays.

[142] Chait R, Ruess J, Bergmiller T, Tkacik G, Guet CC (2017). Shaping bacterial population behavior through computer-interfaced control of individual cells. Nat. Commun..

[143] Cobb, R. E., Sun, N. & Zhao, H. Directed evolution as a powerful synthetic biology tool. *Methods*, **60**, 81–90, 2013. 10.1016/j.ymeth.2012.03.009 PMC3399045 22465795

[144] Goldman N (2013). Towards practical, high-capacity, low-maintenance information storage in synthesized DNA. Nature.

[145] Dvorák P, Nikel PI, Damborsky J, de Lorenzo V (2017). Bioremediation 3.0: engineering pollutant-removing bacteria in the times of systemic biology. Biotechnol. Adv..

[146] TerAvest MA, Li Z, Angenent LT (2011). Bacteria-based biocomputing with cellular computing circuits to sense, decide, signal, and act. Energy Environ. Sci..

[147] Chen YY, Smolke CD (2011). From DNA to targeted therapeutics: bringing synthetic biology to the clinic. Sci. Transl. Med..

[148] Paton RC (1993). Some computational models at the cellular level. BioSystems.

[149] Niederholtmeyer H (2015). Rapid cell-free forward engineering of novel genetic ring oscillators. eLife.

[150] Swank Z, Laohakunakorn N, Maerkl SJ (2019). Cell-free gene-regulatory network engineering with synthetic transcription factors. Proc. Natl. Acad. Sci. USA.

[151] Lehr, F. X. et al. Cell-free prototyping of AND-logic gates based on heterogeneous RNA activators. *ACS Synth. Biol.* **8**, 2163–2173, 2019. 10.1021/acssynbio.9b00238 31393707

[152] Lambert N (2013). Quantum biology. Nat. Phys..

[153] Adriana M (2018). The future of quantum biology. J. R. Soc. Interface.

[154] Engel GS (2007). Evidence for wavelike energy transfer through quantum coherence in photosynthetic systems. Nature.

[155] R. K. Allemann & Scrutton, N.S. *Quantum Tunnelling in Enzyme-Catalysed Reactions* (Royal Society of Chemistry, 2009).

[156] David D, Roger P (1985). Quantum theory, the Church–Turing principle and the universal quantum computer. Proc. R. Soc. Lond. A. Math. Phys. Sci..

[157] Scheres B, Van Der Putten WH (2017). The plant perceptron connects environment to development. Nature.

[158] DeMarseT.B. & Dockendorf, K. P. Adaptive flight control with living neuronal networks on microelectrode arrays. In *Proceedings of the IEEE International Joint Conference on Neural Networks*, vol 3, 1548–1551 (IEEE, 2005).

[159] Warwick, K., Nasuto, S. J., Becerra, V. M. & Whalley, B. J. Experiments with an in-vitro robot brain. In *Computing with Instinct*, 1–15 (Springer, 2011).

[160] Adamatzky, A. *Advances in Physarum machines: Sensing and Computing with Slime Mould*, vol 21 (Springer, 2016).

[161] Tschirhart T (2017). Electronic control of gene expression and cell behaviour in *Escherichia coli* through redox signalling. Nat. Commun..

[162] Abelson H (2000). Amorphous computing. Commun. ACM.

[163] Gordana D.-C. The info-computational nature of morphological computing (ed. Müller, V. C.), *Philosophy and Theory of Artificial Intelligence*, Studies in Applied Philosophy, Epistemology and Rational Ethics, 59–68 (Springer Berlin Heidelberg, Berlin, Heidelberg, 2013).

[164] Umedachi T, Takeda K, Nakagaki T, Kobayashi R, Ishiguro A (2010). Fully decentralized control of a soft-bodied robot inspired by true slime mold. Biol. Cybern..

[165] Solé R (2015). Bioengineering the biosphere?. Ecol. Complex..

[166] Armstrong R (2010). Systems architecture: A new model for sustainability and the built environment using nanotechnology, biotechnology, information technology, and cognitive science with living technology. Artif. Life.

